# Evaluation of Garlic Cultivars for Polyphenolic Content and Antioxidant Properties

**DOI:** 10.1371/journal.pone.0079730

**Published:** 2013-11-13

**Authors:** Shuxia Chen, Xiaoqing Shen, Siqiong Cheng, Panpan Li, Junna Du, Yanxia Chang, Huanwen Meng

**Affiliations:** 1 College of Horticulture, Northwest A&F University/Key Laboratory of Horticultural Plant Germplasm Resources Utilization in Northwest China, Yangling, China; 2 College of Veterinary Medicine, Northwest A & F University, Yangling, China; University of Sassari, Italy

## Abstract

Two phenolic compound parameters (total phenolic and flavonoid contents) and 5 antioxidant parameters (DPPH [2, 2-diphenyl-1-picrylhydrazyl] radical scavenging activity, HRSC (hydroxyl radical scavenging capacity), FRAP (ferric ion reducing antioxidant power), CUPRAC (cupric ion reducing antioxidant capacity), and MCA (metal chelating activity) were measured in bulbs and bolts of 43 garlic cultivars. The bulbs of cultivar ‘74-x’ had the highest phenolic content (total phenolic, flavonoids) and the strongest antioxidant capacity (DPPH, FRAP, and CUPRAC), followed by bulbs of cultivar ‘Hanzhong purple’; the bulbs of cultivar ‘Gailiang’ had the lowest phenolic content and antioxidant capacity (FRAP, CUPRAC, MCA). The bolts of ‘Hanzhong purple’ also had higher phenolic content. Principal components analysis (PCA) separated the cultivars into 3 groups according to phenolic and flavonoid contents and strength of antioxidant activity. The first group had higher HRSC, FRAP, and flavonoid content; the second group had higher total phenolic content and MCA; some cultivars in the third group had higher HRSC and FRAP. All 8 test garlic bulb extracts successfully prevented Human Vascular Endothelial Cell death and significantly prevented reactive-oxygen species (ROS) formation in oxidative stress model, in which cultivar ‘74-x’ had highest protection capability, following by cultivar ‘Hanzhong purple’, and the bulbs of cultivar ‘No. 105 from Korea’ had the lower protection capability against cell death and ROS formation. The protection capability in vivo of these garlic cultivars was consistent with their phenolic content and antioxidant capacity.

## Introduction

Garlic (*Allium sativum* L.) is one of the most commonly produced vegetables worldwide. According to the United Nations Food and Agriculture Organization (FAO), approximately 10 million metric tons of garlic is produced annually on approximately 1 million hectares (2.5 million acres) of land. China is by far the largest producer of garlic, producing over 75% of world tonnage. Garlic is a source of various biologically active phytomolecules, including organosulfur compounds, phenolic acids, allyl thiosulfinates, flavonoids, and vitamins. The health properties of garlic depend on its bioactive compounds and especially on phenolic compounds [1], [2], which have interesting pharmacological properties, are present in relatively high amounts [3]. Many researches have been conducted to assess the dietary role of polyphenolic substances, and their characteristics, metabolic pathways, and biological effects [4], [5], so garlic has been widely used to scavenge Reactive oxygen species (ROS) [6] and treat a variety of diseases including heart disease and cancer [7]. The extract of garlic has significant antioxidant activity and protective effects against oxidative DNA damage [8], decreasing fibrinogen and increasing antioxidant activity in the plasma of rats [9] and reducing the radiation sensitivity of normal tissues that are adjacent to tumors [10], so the extract might be useful in preventing endothelial dysfunction [11]. Garlic is thus widely used to protect humans against oxidative stress [12], reduce the risk of chronic diseases [13], prevent disease progression, and treat or prevent atherosclerosis [7], [14], [15] and cancer [14].

Interest in natural antioxidants and particularly in dietary antioxidants, which are present in vegetables and contribute to protection against oxidative stress in humans, is increasing. Garlic possesses potential health-promoting effects due to its high phenolic phytochemical content and is a source of natural antioxidants [16]. The total phenolic acid content of a local garlic cultivar grown near Namhae-gun, Korea was 17.86 mg/kg of dry matter (dm) and the total flavonoid content was 29.95 mg/kg dm [4]. The total phenolic content varied from 3.4 mg gallic acid equivalents (GAE)/g dm to 10.8 mg GAE/g dm in different garlic cultivars grown at four locations in Andalusia, Spain [3]. The content of phenolic compounds in garlic thus varies greatly with genetic, agronomic, and environmental factors [17], and it is well known that cultivar is the primary factor that determines this variation. Bulb firmness, pH, soluble solids, moisture content, and sugar content differed across 14 garlic cultivars [18]; other traits that vary across cultivars when grown under the same environmental conditions include the leaf number before bolting, flowering date, final stem length, flower/topset ratio, and pollen viability [19]. Variation in allicin, allyl methyl thiosulfinate, and allyl *trans*-1-propenyl thiosulfinate has also been observed in 93 garlic ecotypes [20]. Although some studies have investigated phenolic compounds and antioxidant activity in garlic, very few reports have compared the distribution of phenolic compounds among garlic cultivars in China, or between garlic bulbs and bolts among these cultivars. It is necessary to assess this trait in garlic germplasm resources to assure their effective utilization [21]–[24].

This study was designed to determine the variability in total phenolics, flavonoids, and antioxidant capacity among bulbs and bolts of 43 garlic cultivars for further development and utilization of these cultivars.

## Materials and Methods

### Materials

The 43 cultivars employed in this study were selected from the garlic germplasm nursery of Northwest A&F University College of Horticulture, Yangling, China; 40 of the cultivars were introduced from China, while 1 was from Ethiopia, 1 was from Thailand, and 1 was from Korea. The numeric identifiers of these 43 major garlic cultivars are shown in Table 1. The cultivars were planted in the garlic germplasm nursery, Northwest A&F University. The row spacing was 20 cm, plant spacing was 15 cm, and depth of the seed furrow was 5 cm. Human umbilical vein endothelial cells (ECV-304) were supplied by assistant professor Dong who worked in the College of Veterinary Medicine, Northwest A&F University.

**Table 1:** Numbers and names of 43 major garlic cultivars.

No.	Cultivar	No.	Cultivar	No.	Cultivar	No.	Cultivar
1	Xiangfan ershui early	12	Cangshan	23	83	34	No. 96
2	Wenjiang seven star red	13	Caijiapo seven leaves	24	Xuzhou white	35	No. 97 from Guizhou
3	Peng county early	14	Yao county red	25	Sicuan purple	36	No. 98 from Guizhou
4	Peng county mid-maturity	15	Xiangfan red	26	Ethiopia white	37	No. 99 from Guizhou
5	Peng county late	16	Jiading No. 2	27	87-x	38	No. 100 from Guizhou
6	Putuo	17	Taicang white	28	Hanzhong red	39	No. 101 from Guizhou
7	Baihe early	18	Russian garlic	29	Sicuan red	40	Solyeng Korea
8	Ningqiang mountain garlic	19	Luliang garlic	30	Hanzhong purple	41	Suzhou white
9	Long county early	20	Bijie garlic	31	Japan white	42	Thailand
10	Xingping white	21	74-x	32	Fenggang white	43	No. 105 from Korea
11	Gailiang	22	No. 75	33	No. 95		

### Samples

Fresh bolts of the 32 bolting cultivars (the others were non-bolting cultivars) were sampled and collected in April 2009 and 2010 and the bulbs of all mature garlic cultivars were sampled in July 2009 and 2010 after air drying in the field. For each cultivar, samples were prepared by mixing equal amounts of garlic powder from 5 bulbs and 10 bolts selected randomly. The garlic cloves or bolts were manually peeled and immediately ground into powder in liquid nitrogen. The powder was stored at −70°C until analysis.

Garlic bulbs of eight cultivar were choosed randomly for testing the cytoprotection against oxidative stress cell death and ROS formation after preparation of phenolic extracts, which were cultivar ‘Cangshan’, ‘74-x’, ‘Hanzhong purple’, ‘Suzhou white’, ‘Ningqiang mountain garlic’, ‘Russian garlic’, ‘No. 97’ and ‘No. 105 from Korea’.

### Chemicals

Folin-Ciocalteu reagent, crystalline aluminum chloride, sodium nitrite, anhydrous sodium carbonate, methanol, hydrochloric acid, and 2, 2-diphenyl-1-picrylhydrazyl (DPPH) were purchased from Tianjin Bodi Chemical Co. (Tianjin, China). The following standards were purchased from Sigma-Aldrich Chemical Co. (Shanghai, China): gallic acid, rutin, neocuproine, brown alloxazine, and 6-hydroxy-2, 5, 7, 8-tetramethylchroman-2 -carboxylic acid (Trolox), Dulbecco's modified Eagle's medium (DMEM), Fetal bovine serum (FBS), trypan blue, glyoxal, methylglyoxal or tertiary-butyl hydroperoxide and all other chemicals were of the highest quality commercially available and were purchased from Sigma–Aldrich Chemical Co. (Shanghai, China).

### Preparation of phenolic extracts

The garlic bulbs and bolts were first treated using the method described by Park et al. [8] with minor modifications. Raw garlic was freeze-dried after steam blanching and was soaked in distilled water in a sealed flask for 14 d in a 70°C water bath. The extracts were centrifuged at 4000× g for 10 min at 4°C using a Sorvall RC-5C Plus centrifuge (Kendro Laboratory Products, Newton, CT, USA). The supernatants were stored at −20°C in the dark until use.

For testing cytotoxicity and ROS formation in vitro, the phenolic extracts were prepared from garlic bulbs and the extracts were freeze-dried in the freeze dryer (FD8505, Sigma-Aldrich Chemical Co., Shanghai, China) and then the phenolic extracts concentrations were adjusted to 1 mg/mL.

### Photometric determination of total phenolic content (TPC) and total flavonoid content (TFAC)

Total phenolic content was determined in the bulbs and bolts of garlic cultivars using the Folin-Ciocalteu method [25] and a Shimadzu UV-1700 analyzer (Shimadzu Co.). Garlic extracts (100 µL) were diluted using 5.9 mL water and then mixed. Next, 200 µL Folin-Ciocalteu reagent was added into the mixture, and 2 mL sodium carbonate solution was added 1 min later. The mixture was allowed to react at room temperature in the dark for 120 min, and then the absorbance was measured at 735 nm. Gallic acid was used as a standard, and the results were expressed as milligrams of gallic acid equivalents (GAE) per gram of garlic fresh weight (FW).

The total flavonoid content (TFAC) was determined according to the method of Sellappan et al. [26] with some modifications. One milliliter of garlic extract was added to 200 µL of 0.5 mol/L sodium carbonate solution. The mixture was allowed to stand for 5 min and was then added to 200 µL of 0.3 mol/L aluminum chloride crystalline and incubated for an additional 5 min. Then, 1.0 mL NaOH (1 M) was added to the reaction system and the absorbance was measured against a blank at 510 nm. The results were calculated and expressed as micrograms of rutin equivalents (RAE) per gram dry weight (DW).

### Measurement of antiradical properties

We followed a previously described method for measuring DPPH free radical-scavenging capacity [27], [28], with minor revisions. First, 100 µL garlic extracts were diluted with 900 µL water. DPPH methanolic solution was then added and the mixture was kept in the dark for 30 min. The absorbance at 515 nm was recorded to determine the concentration of remaining DPPH, and the results were expressed as micromoles of Trolox equivalents per gram of garlic mass.

The percentage inhibition of DPPH of the test sample and known solutions of Trolox were calculated by the following formula:
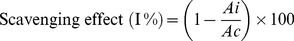
(1)


Where *Ai* is the absorbance of the sample at 515 nm, and *Ac* is the absorbance of the blank at 515 nm.

HRSC was estimated by the methods of Prasad et al. [13]. Briefly, 3 mL distilled water and 100 µL FeSO_4_ (0.02 M) were added to a microfuge tube. Next, 45 µL H_2_O_2_ (0.15%) solution and 1 mL SA (8 M) were added. The final volume of the reaction mixture was then added to 100 µL sample solution and kept in the dark for 50 min at 20°C. The absorbance at 510 nm was recorded, and HRSC was calculated as follows:
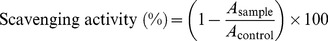
(2)


FRAP was measured as previously described [29] with minor modifications. First, 100 µL sample, 2.5 mL phosphate buffer (0.2 M, pH 6.6), and 2.5 mL potassium ferricyanide solution (1%) were sequentially mixed, and the mixture was then incubated in a 50°C water bath for 20 min before cooling. Next, 2.5 mL potassium ferricyanide (1%) was added, and the mixture was incubated in a 50°C water bath for 20 min. After cooling, 2.5 mL 3-chloroacetic acid (10%) was added and the solution was mixed. Then, 2.5 mL the mixture was extracted into a new tube, and 2.5 mL distilled water was added. Finally, 300 µL FeCl_3_ (0.1%) was added to this mixture and the reaction was allowed to proceed for 5 min at room temperature in the dark. The absorbance of the product was measured at 700 nm; FRAP was expressed as this absorbance.

CUPRAC was determined using previously described methods [30], with minor modifications. Briefly, 0.1 mL garlic extract was mixed with 1 mL CuSO_4_ (5 mM), 1 mL neocuproine (3.75 mM), 1 mL ammonium acetate (1 mM), and 1 mL distilled water and kept in the dark for 30 min in a 37°C water bath. Absorbance was then measured at 450 nm. Results are expressed in milligrams of Trolox per liter of extract.

Metal chelating capacity was measured using the methods of Prakash et al. [29]. One-hundred microliters of extract was mixed with 3.9 mL distilled water, 100 µL FeCl_2_⋅4H_2_O (2 mM), and 50 µL ferrozine (5 mM). The reaction mixture was kept in the dark for 10 min and the Fe^2+^ concentration was then monitored at 562 nm. The percentage chelating capacity was expressed as follows:
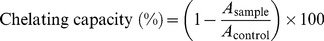
(3)


Where *A* is absorbance at 562 nm.

ECV-304 viability was tested microscopically by plasma membrane disruption, as determined by the trypan blue (0.1% w/v) exclusion test [31]. Human Vascular Endothelial Cell viability was assessed at 3 hours, and the cells were at least 90% viable before use.

ECV-304 reactive-oxygen species (ROS) generation induced by tertiary butyl- hydroperoxide was determined by adding dichlorofluorescin diacetate (DCFH-DA) to the Human Vascular Endothelial Cell. DCFH-DA penetrated cell and was hydrolyzed to form non-fluorescent dichlorofluorescin (DCFH). DCFH was then oxidised by ROS to form the highly fluorescent dichlorofluorescein (DCF). After incubation with tertiary butyl hydroperoxide, 1 mL samples of cell were withdrawn at 3 hours and centrifuged at 1000× g for 5 minute. The cells were resuspended in DMEM and 10 µM DCFH-DA was added. Cells were allowed to incubate at 37°C for 20 minutes. The fluorescent intensity of DCF was determined by VictorTM X5 multilabel reader (PerkinElmer Life and Analytical Sciences) measuring the excitation and emission wavelengths, which were 490 and 520 nm, respectively.

### Statistical analysis

All data are expressed as the mean ±SD of 3 replicates, and statistical analyses were subjected to ANOVA procedures (DPS v7.55 for Windows). Significant differences among cultivars were detected by Duncan's multiple range tests. *P*-values <0.05 were considered significant.

Frequency distribution histograms of polyphenolic content were constructed according to the distribution interval among cultivars, where the interval was equal to the Value_maximum_ minus the Value_minimum_ divided by N [32].

Principal component analysis (PCA) was used to detect clustering and to establish relationships between cultivars and polyphenolic content and antioxidant properties. To eliminate the influence of dimension, data were classified into 10 grades for analysis: grade 1<X−2δ and grade 10>X+2δ, where the interval of each grade was 0.5δ, and δ was the standard deviation.

## Results and Discussion

### The distribution of garlic polyphenolic content and antioxidant properties among different cultivars

**Figure 1:**
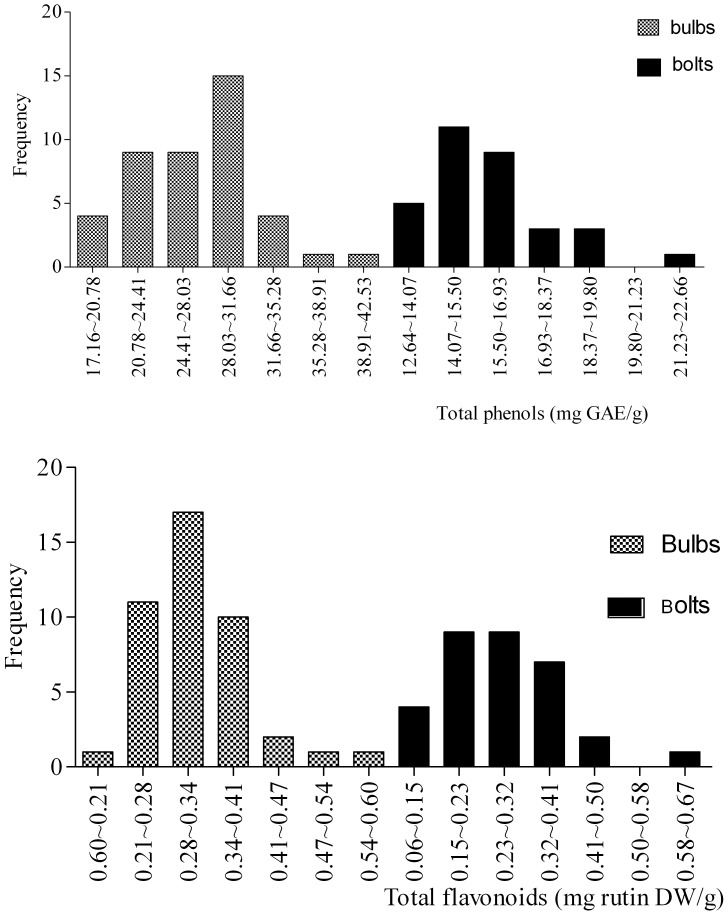
Frequency distribution of total phenolic (A) and flavonoid (B) contents in garlic bulbs and bolts of different cultivars.

The frequency distributions of TPC and TFAC among bulbs and bolts of different garlic cultivars are shown in Fig. 1. TPC in bulbs exhibited a partially normal distribution and ranged from 21.27 to 33.96 mg GAE/g FW in most cultivars (Fig.1A). Previous studies have shown TPC values of 5.63 mg GAE/g in aged garlic extract [3], [8] and 15.23 mg GAE/g FW [32], which are much lower than the TPC observed in our 43 garlic cultivars. However, TPC in bolts of 78.1% of hardneck garlic cultivars was much lower than that in bulbs, ranging from 9.24 to 15.48 mg GAE/g, and these results are in accordance with those of Nuutila et al. [16], who detected a TPC of 0.075–0.080 mg GAE/g in different *Allium* species.

The TFAC in both bulbs and bolts exhibited an almost normal distribution, ranging from 0.11 to 0.59 mg rutin DW/g FW and 0.075 to 0.675 mg rutin DW/g FW, respectively(Fig.1B). There was no significant difference in TFAC between the bulbs and bolts of different cultivars.

### Polyphenolic content and antioxidant properties of different cultivars

**Figure 2:**
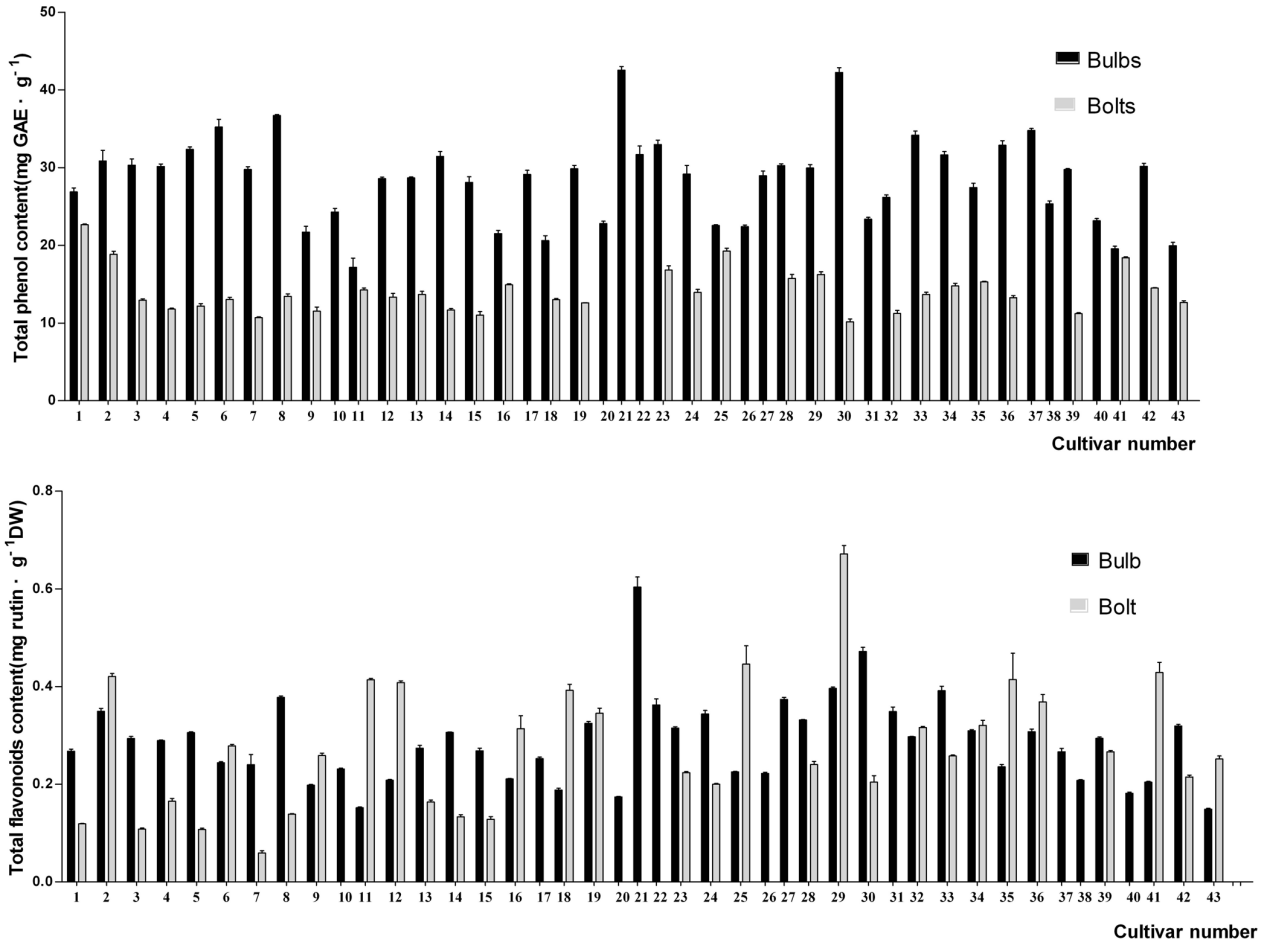
Total phenol content (A) and total flavonoid content (B) of bulbs and bolts in different garlic cultivars. Numbers identifying the garlic cultivars are the same as in Table 1. Averages and standard deviations of biological triplicates are shown.

The extracts of bulbs from over 40 cultivars were evaluated for total phenolic content and antioxidant activities to determine the extent of genotypic variation. The coefficient of variation in TPC in bulbs and bolts was 3.06%, indicating that the TPC varied significantly among different cultivars (Fig. 2). TPC in bulbs of the 43 garlic cultivars varied from 17.16 to 42.53 mg GAE/g. Bulbs of cultivar ‘74-x’ had the highest TPC, followed by the ‘Hanzhong purple’ cultivar; cultivar ‘Gailiang’ had the lowest TPC. These results suggest that cultivar ‘74-x’ may be a better source of phenolic compounds than other garlic cultivars (Fig. 2A).

The TFAC in bulbs also varied significantly (more than 4-fold) among cultivars (Fig. 2B), with a coefficient of variation of 2.70%. TFAC ranged from 0.15 (cultivar ‘No. 105 from korea’) to 0.60 mg rutin DW/g (cultivar ‘74-x’ respectively). Cultivar ‘74-x’ also had the highest TFAC in bulbs of the 43 cultivars, followed by the cultivar ‘Hanzhong purple’, while cultivars ‘No. 105 from korea’ and ‘Gailiang’ had the lowest TFAC. This result was consistent with that of TPC.

Variability of TPC and TFAC in bulbs of different cultivars could be attributed to various cultivar characteristics. Clove size must be considered, as it indirectly affects the final concentration of phenolic compounds. In agreement with our results, previous reports have shown that different garlic cultivars had different yields [33], allicin content [21], and polyphenolic content [32], although Sterling and Eagling [34] reported that the variety and origin of garlic were not important factors affecting the agriculture traits of this crop.

TPC in garlic bolts of 32 cultivars varied from 10.17 mg GAE/g (cultivar ‘Xiangfan ershui early’) to 22.66 mg GAE/g (cultivar ‘Hanzhong purple’), which was much higher than that in leaves of *Allium roseum* L., where the polyphenol content was 1.23 mg GAE/g aged extract [35]; the TPC in bulbs was higher than that in bolts. In these 32 cultivars, TFAC in garlic bolts varied from 0.06 to 0.67 mg rutin DW/g (cultivars ‘Baihe early’ and ‘Sicuan red’ respectively) ‘Hanzhong purple’, which had the highest TPC also had a higher TFAC. The coefficients of variation for TPC and TFAC were 3.47% and 5.85%, respectively.

Normally, organisms have several different antioxidant systems that may be involved in various interactions; thus, the methods used to analyze antioxidant activity should accurately reflect all of the antioxidants in a complex system. In this study, 5 in vitro assays (DPPH, HRSC, FRAP, CUPRAC, and metal chelating capacity) were used as complementary methods to evaluate the potential antioxidant activity of garlic cultivars. Significant differences were observed among different cultivars in these assays (Table 2). Among the methods available, those based on the elimination of free radicals, particularly on scavenging of DPPH radicals have been used most frequently. The DPPH-scavenging activity of garlic bulbs ranged from 3.60% to 45.63%, which was exceeded the range (5.07% to 11.36%) reported for the *Allium* genus [35]. In contrast, DPPH values were much higher (66.48%) in the leaves of *A. sativum* L. and in fried garlic (60.85%) [6].

**Table 2:** Analysis of significant differences in antioxidant activity in bulbs and bolts from different garlic cultivars.

Cultivar	Bulbs					Bolts				
	DPPH	·OH	FRAP	CRAC	MCA	DPPH	FRAP	·OH	CRAC	MCA
1	4.82±3.01 (stu)	60.04±3.78 (n)	0.39±0.01 (jklmn)	4058.71±409.30 (lmnopqrst)	11.69±4.42 (fghijklm)	39.39±1.30 (bcd)	46.27±9.58 (a)	0.40±0.01 (a)	6287.70±184.56 (a)	5.97±2.72 (defg)
2	23.80±4.25fghijk	80.02±0.69 (abc)	0.44±0.02 (defg)	4654.71±355.67 (efghijklmn)	18.11±5.75 (abcde)	32.64±1.20 (fg)	35.24±3.89 (cdefghi)	0.33±0.01 (b)	5561.51±254.59 (b)	5.48±3.56 (defg)
3	14.52±2.10 (lmnopq)	62.16±1.05 (lmn)	0.48±0.01 (bcde)	4577.75±210.68 (ghijklmno)	7.93±1.49 (klmnopq)	9.94±1.24 (mn)	40.60±4.03 (abcdef)	0.24±0.00 (klm)	3409.27±57.63 (pq)	7.92±4.40 (cdefg)
4	16.08±2.49 (klmno)	66.11±1.97 (ijklmn)	0.44±0.11 (defg)	4820.60±590.62 (defghijkl)	8.47±6.68 (jklmnopq)	16.42±1.73	38.35±4.50 (abcdefg)	0.25±0.01 (ijk)	3428.74±71.06 (opq)	6.75±6.92 (cdefg)
5	32.02±2.93 (bcdef)	37.87±5.87 (p)	0.44±0.01 (defg)	5263.26±302.86 (bcdefg)	8.26±4.19 (jklmnopq)	17.40±2.05 (j)	42.96±5.10 (abc)	0.26±0.01 (ij)	3456.00±136.40 (opq)	10.40±9.62 (bcdefg)
6	9.67±6.65 (opqrstu)	72.46±5.53 (cdefghi)	0.39±0.04 (ijklm)	4098.60±406.98 (lmnopqrs)	14.84±4.40 (cdefghi)	34.83±2.27 (ef)	30.83±0.78 (ghijk)	0.26±0.00 (hij)	4094.63±30.91 (hij)	4.83±3.90 (defg)
7	4.30±3.41 (tu)	65.72±0.67 (ijklmn)	0.41±0.01 (ghijk)	4531.25±324.07 (ghijklmno)	12.65±4.44 (efghijkl)	10.12±0.95 (mn)	38.25±1.60 (abcdefg)	0.23±0.01 (mn)	3335.34±73.11 (q)	9.28±5.72 (cdefg)
8	34.55±0.91 (bcd)	76.63±1.13 (abcdef)	0.48±0.01 (bcde)	5414.84±569.47 (bcde)	5.75±3.16 (mnopqr)	16.48±1.86 (jk)	42.38±3.43 (abcd)	0.27±0.01 (fghi)	3609.18±52.19 (mno)	4.52±4.02 (defg)
9	16.72±3.03 (jklmno)	62.07±3.35 (lmn)	0.36±0.01 (mnopq)	4255.11±799.48 (ijklmnopq)	16.26±3.01 (bcdefg)	21.37±0.96 (i)	20.42±15.23 (mn)	0.24±0.00 (lm)	3751.95±172.22 (lm)	1.49±0.77 (g)
10	23.94±3.70 (fghijk)	66.66±2.58 (hijklmn)	0.30±0.03 (stu)	3894.68±521.73 (nopqrstu)	7.18±3.55 (lmnopqr)	NB	NB	NB	NB	NB
11	13.81±14.12 (lmnopqr)	75.78±2.65 (abcdef)	0.27±0.01 (u)	3350.69±127.31 (rstu)	22.98±2.51 (a)	24.10±1.61 (i)	19.66±9.03 (n)	0.29±0.01 (de)	4250.39±82.05 (gh)	3.78±3.41 (defg)
12	5.12±5.41 (rstu)	62.12±7.23 (lmn)	0.33±0.02 (opqrs)	3545.71±450.23 (qrstu)	14.22±5.19 (defghij)	31.49±0.72 (gh)	47.02±5.73 (a)	0.23±0.00 (mno)	3297.92±38.19 (qr)	7.22±8.35 (cdefg)
13	5.46±0.38 (rstu)	63.41±1.28 (klmn)	0.37±0.01 (lmno)	3857.95±642.12 (opqrstu)	9.74±5.24 (hijklmno)	15.27±1.27 (jkl)	43.15±1.14 (abc)	0.28±0.01 (efg)	3685.75±101.17 (mn)	6.91±5.69 (cdefg)
14	24.22±1.69 (fghijk)	61.54±3.50 (mn)	0.51±0.01 (b)	4529.39±815.60 (ghijklmno)	8.50±5.69 (jklmnopq)	13.06±2.43 (lm)	41.84±3.15 (abcde)	0.24±0.02 (klm)	3126.93±96.37®	12.88±8.28 (bcd)
15	6.39±1.18 (qrstu)	69.75±2.33 (fghijkl)	0.44±0.01 (efgh)	4616.13±696.02 (fghijklmno)	9.07±2.90 (ijklmnop)	8.63±2.63 (n)	33.75±2.84 (defghij)	0.21±0.01 (opq)	2918.65±96.96	10.60±7.43 (bcdefg)
16	7.27±10.18 (pqrstu)	78.51±2.51 (abcde)	0.36±0.02 (lmno)	4013.89±650.00 (mnopqrst)	21.35±3.53 (ab)	40.41±2.47 (bcd)	36.94±1.93 (bcdefgh)	0.29±0.01 (ef)	4554.40±53.04 (f)	10.18±0.96 (bcdefg)
17	15.52±6.31 (klmnop)	62.25±6.37 (lmn)	0.36±0.04 (lmnop)	4232.77±533.79 (ijklmnopq)	17.40±5.26 (abcdef)	NB	NB	NB	NB	NB
18	10.06±2.35 (opqrstu)	77.20±0.63 (abcdef)	0.35±0.01 (nopqr)	4122.30±435.73 (klmnopqr)	19.78±0.94 (abcd)	13.96±3.74 (kl)	25.46±1.90 (jklmn)	0.22±0.02 (nop)	3882.94±123.91 (kl)	1.50±1.60 (g)
19	25.75±2.88 (efghi)	47.03±3.82 (o)	0.40±0.00 (hijkl)	4724.15±359.05 (efghijklm)	5.56±1.59 (nopqr)	9.25±4.50 (n)	29.39±4.50 (ghijklm)	0.23±0.00 (mn)	3426.59±79.27 (pq)	3.03±2.60 (efg)
20	9.07±4.99 (opqrstu)	41.87±13.60 (op)	0.31±0.03 (rstu)	3200.40±369.88 (u)	16.19±3.16 (bcdefg)	NB	NB	NB	NB	NB
21	45.63±1.81 (a)	75.61±3.39 (abcdefg)	0.61±0.00 (a)	8305.43±990.499 (a)	2.92±2.83 (qr)	NB	NB	NB	NB	NB
22	33.77±0.84 (bcde)	61.14±6.50 (mn)	0.44±0.00 (defg)	5731.03±365.42 lpar;bc)	2.91±1.99 (qr)	NB	NB	NB	NB	NB
23	37.80±1.33 (abc)	73.46±3.11 (bcdefghi)	0.41±0.01 (ghijk)	5392.46±802.82 (bcdef)	2.51±0.99 (qr)	46.09±2.98 (a)	27.15±7.57 (ijklmn)	0.33±0.02 (b)	4938.49±96.23 (c)	8.76±6.41 (cdefg)
24	24.91±4.76 (fghij)	46.51±17.01 (o)	0.45±0.02 (cdefg)	3872.66±149.15 (nopqrstu)	15.70±2.51 (bcdefgh)	40.83±1.53 (bc)	32.33±4.28 (fghijk)	0.26±0.01 (ghij)	4208.57±72.83 (gh)	11.01±1.68 (bcde)
25	8.82±5.11 (opqrstu)	64.30±3.87 (jklmn)	0.32±0.02 (pqrs)	4141.82±292.22 (jklmnopqr)	17.32±2.69 (abcdef)	39.09±2.08 (bcd)	45.26±3.91 (ab)	0.40±0.02 (a)	4815.48±171.69 (cd)	15.28±3.51 (abc)
26	4.84±3.90 (stu)	80.32±4.82 (ab)	0.32±0.02 (qrst)	3295.63±411.07 lpar;tu)	20.24±4.11 (abcd)	NB	NB	NB	NB	NB
27	9.95±6.95 (opqrstu)	81.31±0.92 (a)	0.43±0.03 (fghi)	4195.08±682.99 (ijklmnopq)	13.73±4.77 (efghijk)	NB	NB	NB	NB	NB
28	19.77±5.52 (hijklm)	73.99±1.51 (abcdefgh)	0.43±0.01 (fghi)	4740.08±69.76 (efghijklm)	11.26±3.30 (ghijklmn)	47.33±1.51 (a)	33.11±6.26 (efghij)	0.31±0.01 (c)	4618.35±18.24 (ef)	23.43±14.38 (a)
29	27.52±0.65 (defghi)	77.71±2.67 (abcde)	0.39±0.02 (jklmn)	5102.37±411.18 (cdefgh)	1.71±0.74 (r)	16.09±0.55 (jkl)	29.52±10.06 (ghijkl)	0.27±0.00 (ghij)	4339.95±50.92 (g)	4.73±4.65 (defg)
30	12.76±7.07 (mnopqrst)	75.68±4.00 (abcdef)	0.61±0.01 (a)	5145.83±404.06 (bcdefgh)	8.22±5.87 (jklmnopq)	4.82±1.00 (o)	24.85±2.60 (jklmn)	0.20±0.00 (q)	3292.06±129.90 (qr)	2.41±1.92 (efg)
31	29.36±1.85 (cdefg)	77.65±3.28 (abcde)	0.43±0.00 (fghij)	3876.56±438.56 (nopqrstu)	16.30±3.73 (bcdefg)	NB	NB	NB	NB	NB
32	13.30±10.76 (lmnopqrs)	79.88±0.46 (abc)	0.45±0.01 (cdef)	5562.69±529.75 (bcd)	22.93±1.60 (a)	0.98±1.41 (p)	23.88±0.90 (klmn)	0.21±0.01 (pq)	3537.77±66.43 (nop)	1.65±0.86 (fg)
33	38.47±0.62 (ab)	63.23±1.84 (klmn)	0.47±0.03 (bcde)	5900.95±575.67 lpar;b)	2.77±3.23 (qr)	14.17±0.64 (kl)	20.48±1.77 (mn)	0.25±0.00 (ijk)	3555.69±84.42 (nop)	1.68±2.62 (fg)
34	18.93±2.81 (ijklmn)	75.89±0.98 (abcdef)	0.49±0.02 (bcd)	4963.00±159.47 (cdefghi)	10.43±2.80 (ghijklmno)	42.05±0.87 (b)	25.21±3.59 (jklmn)	0.28±0.02 (efgh)	4321.93±85.04 (g)	10.86±4.16 (bcdef)
35	3.60±1.63 (u)	67.02±3.42 (hijklmn)	0.39±0.01 (ijklm)	4429.92±506.20 (hijklmnop)	7.14±5.59 (lmnopqr)	37.49±3.12 (de)	28.77±6.19 (hijklm)	0.27±0.01 (ghij)	4753.61±120.19 (de)	18.69±10.93 (ab)
36	28.28±3.76 (defgh)	66.54±1.17 (hijklmn)	0.38±0.01 (klmn)	5258.56±502.27 (bcdefg)	3.65±2.17 (pqr)	33.21±1.37 (fg)	27.11±4.42 (ijklmn)	0.25±0.01 (jkl)	3987.81±60.51 (ijk)	11.15±4.96 (bcde)
37	15.94±9.78 (klmnop)	78.75±3.82 (abcd)	0.40±0.03 (hijkl)	4388.76±114.53 (hijklmnop)	11.13±4.97 (ghijklmn)	NB	NB	NB	NB	NB
38	10.55±13.28 (nopqrstu)	69.53±1.83 (fghijkl)	0.39±0.01 (jklmn)	4032.20±470.78 (lmnopqrst)	4.72±4.60 (opqr)	NB	NB	NB	NB	NB
39	21.83±1.17 (ghijkl)	70.75±0.99 (efghijk)	0.49±0.02 (bc)	4909.34±305.42 (defghijk)	5.03±1.26 (opqr)	4.99±0.14 (o)	21.54±0.24 (lmn)	0.20±0.00 (q)	3323.60±222.88 (q)	3.85±0.60 (defg)
40	5.04±4.55 (stu)	67.89±3.65 (ghijklm)	0.28±0.03 (tu)	3683.89±250.10 (pqrstu)	18.30±2.42 (abcde)	NB	NB	NB	NB	NB
41	11.69±5.83 (mnopqrstu)	71.44±2.65 (defghij)	0.36±0.01 (mnopq)	3683.10±579.82 (pqrstu)	18.12±0.46 (abcde)	46.82±1.18 (a)	26.81±5.72 (ijklmn)	0.31±0.01 (cd)	4801.49±60.51 (cd)	5.96±3.44 (defg)
42	20.09±1.04 (hijklm)	61.54±5.46 (mn)	0.43±0.01 (fghij)	4931.25±307.21 (defghij)	13.27±3.64 (efghijk)	28.52±0.33 (h)	36.62±2.37 (bcdefgh)	0.28±0.00 (efg)	3922.62±120.82 (jkl)	7.19±4.88 (cdefg)
43	15.04±6.04 (lmnopq)	76.41±6.06 (abcdef)	0.33±0.01 (opqrs)	3323.78±292.45 (stu)	20.68±1.92 (abc)	37.95±3.01 (cde)	31.15±7.24 (ghijk)	0.26±0.02 (ij)	4160.83±77.78 (ghi)	8.05±6.66 (cdefg)

Notes: DPPH: DPPH radical scavenging activity; ·OH: hydroxyl radical scavenging activity; FRAP: ferric ion reducing antioxidant power; CRAC: cupric ion reducing antioxidant capacity; MCA: metal chelating activity; NB: nonbolting cultivar. Different. lowercase letters are significantly different at *P*<0.05.

Cultivars ‘No. 97 from Guizhou’, ‘Baihe early’, and ‘Xiangfan ershui early’ had relatively low antioxidant activities, while cultivar ‘74-x’, which had the highest TPC among all the cultivars, showed the highest antioxidant activity. The DPPH-scavenging activity of cultivar ‘74-x’ was 12.68-fold higher than that of cultivar ‘No. 97 from Guizhou’. Significant differences in HRSC were found among cultivars; and the strongest scavenging activity (81.31%) was observed in bulbs of cultivar ‘87-x’ and the lowest •OH scavenging capacity (37.87%) was shown in bulbs of cultivar ‘Pengxian late’.

Currently, the most frequently used method for measuring antioxidant activity is FRAP, which is often expressed as absorbance value, i.e., the higher the absorbance, the stronger the reducing capability. Significant differences based on FRAP assays were observed among cultivars. The lowest absorbance value in bulbs was 0.273 (cultivar ‘Gailiang’), and the TPC of this cultivar was also the lowest among all cultivars. The highest absorbance value was 0.613 (cultivar ‘Hanzhong purple’), followed by 0.611 (cultivar ‘74-x’). The reducing capacity of cultivar ‘Gailiang’ was only 44.54% of that of ‘Hanzhong purple’.

In contrast to FRAP, which is based on the ferric-ferrous system, the CUPRAC method is based on the potential to reduce copper ions. We found that cultivar ‘74-x’ possessed the highest reducing activity as measured by CUPRAC (8305.43 µg Trolox DW/g), and cultivar ‘Bijie’ had the lowest reducing activity (3200.40 µg Trolox DW/g; 38.53% that of cultivar ‘74-x’). The metal chelating capacity in bulbs ranged from 1.71% (cultivar ‘Gailiang’) to 22.98% (cultivar ‘Sichuan red’).

Antioxidant activities in bolts differed greatly according to the assay used (Table 2). The DPPH concentration varied from 0.98% (‘Fenggang white’) to 47.33% (‘Hanzhong red’), while •OH scavenging capacity varied from 19.7% (‘Gailiang’) to 47.0% (‘Cangshan’), and FRAP ranged from 0.19632 (‘No. 101 from Guizhou’) to 0.399 (‘Sichuan purple’). The CUPRAC of garlic bolts varied from 2918.65 (‘Xiangfan red’) to 6287.70 µg Trolox DW/g (‘Xiangfan early’), and which of different cultivar ranged from 1.49% (‘Longxian early’) to 23.43% (‘Hanzhong red’).

### PCA of polyphenolic content and antioxidant properties

**Figure 3:**
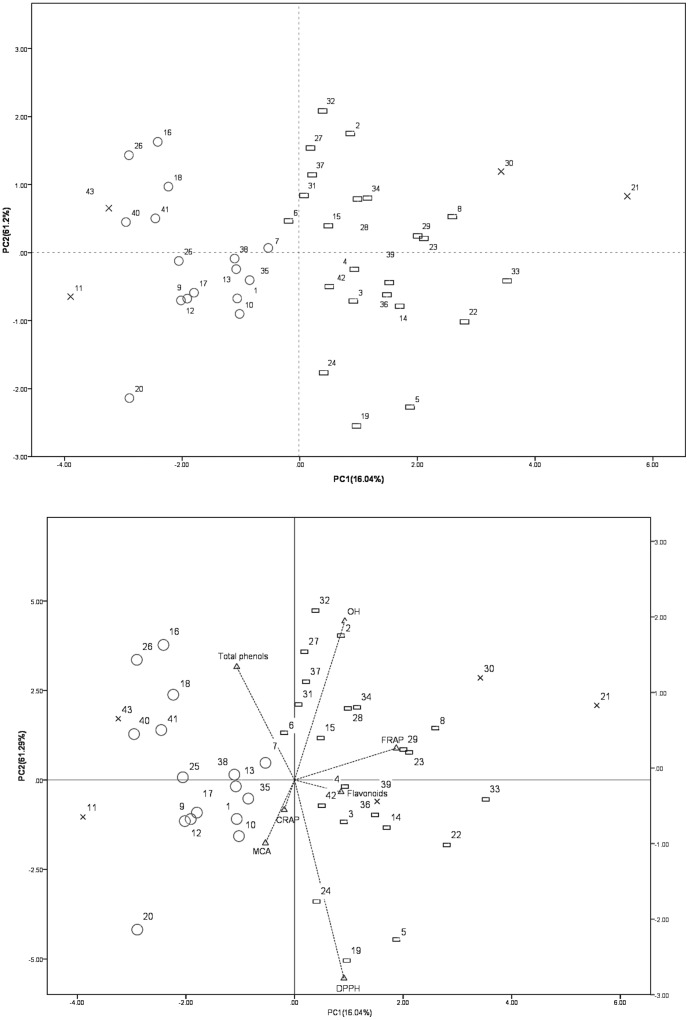
Principal component analysis (PCA) of the investigated garlic cultivar bulbs according to the first 2 PCAs obtained by polyphenolic content and antioxidant properties (DPPH, ·OH, FRAP, CUPRAC, and MCA). (A) Scatter plots of PC1 and PC2 and (B) loadings plot of PC1 and PC2. The numbers in (A) correspond to the sample numbers given in Table 1. Percentages in parentheses are the variance of each component.

PCA allows a large number of variables to be reduced to just a few that accounts for most of the variance in the observed results. Thus, we performed PCA to determine which garlic cultivars were associated with TPC and antioxidant activity in their bulbs (Fig.3). The 43 cultivars could be divided into 3 groups based on their polyphenolic content and antioxidant properties (loadings). The first group contained 23 cultivars: ‘Wenjiang seven star red’, ‘Peng county early’, ‘Peng county mid-maturity’, ‘Peng county late’, ‘Putuo’, ‘Ningqiang mountain garlic’, ‘Yao county red’, ‘Xiangfan red’, ‘Luliang garlic’, ‘No. 75’, ‘No. 83’, ‘Xuzhou white’, ‘87-x’, ‘Hanzhong red’, ‘Sicuan red’, ‘Japan white’, ‘Fenggang white’, ‘No. 95’, ‘No. 96’, ‘No. 98 from Guizhou’, ‘No. 101 from Guizhou’, and ‘Thailand qingmai’; the second group contained 16 cultivars: ‘Xiangfan ershui early’, ‘Baihe early’, ‘Long county early’, ‘Xingping white’, ‘Cangshan’, ‘Caijiapo seven leaves’, ‘Jiading No. 2’, ‘Taicang white’, ‘Russian garlic’, ‘Bijie garlic’, ‘Sicuan purple’, ‘Ethiopia white’, ‘No. 97 from Guizhou’, ‘No. 99 from Guizhou’, ‘No. 100 from Guizhou’, ‘Solyeng Korea’, and ‘Suzhou white’; and the third group included 3 cultivars: ‘Gailiang’, ‘74-x’, ‘Hanzhong purple’, and ‘No. 105 from Korea’. Scatter-plot scores of the PCA for the cultivars and the corresponding loadings plots are shown in Fig. 3. The first 3 PCs contained a large amount of important information and accounted for 86% of the total variance.

The higher positive value of PC1 showed that some cultivars (‘74-x’, ‘No. 75’, ‘87-x’, ‘Hanzhong purple’, and ‘No. 95’) had stronger hydroxyl radical scavenging activity, FRAP, and higher flavonoid contents. The lower negative value of PC1 showed that some cultivars (‘No. 95’, ‘Bijie garlic’, ‘Ethiopia white’, ‘No. 105 from Korea’, and ‘Solyeng Korea’) had higher TPC. The higher positive value of PC2 showed that a number of cultivars, including ‘Wenjiang seven star red’, ‘Jiading No. 2’, ‘Ethiopia white’, ‘87-x’, and ‘Fenggang white’, had stronger hydroxyl radical scavenging activity, FRAP, and higher TPC. The lower negative value of PC2 showed that cultivars, including ‘Xingping white’, ‘Luliang garlic’, and ‘Xuzhou white’, had higher MCA, DPPH, CUPRAC and flavonoid content (Fig 3B).

Almost all of cultivars in the first group had the higher positive value of PC1 so it indicated that the first group had higher HRSC, FRAP and flavonoid content. The second group had lower negative value of PC1 so it indicated that the second group had higher total phenolic content and MCA. Some cultivars such as ‘74-x’ and ‘Hanzhong purple’ in the third group had higher HRSC, FRAP and flavonoid content.

### Correlation between phenolic compounds and antioxidant capacity

Correlation analyses were performed among the phenolic compounds and antioxidant capacity parameters, and between each phenolic compound and antioxidant capacity measurement for the 43 cultivars. These analyses showed significant positive correlations between TPC and TFAC, and among DPPH, FRAP, and CUPRAC in both bulbs (Table 3) and bolts (Table 4). There were also significant correlations among DPPH, FRAP, and CUPRAC antioxidant assay measurements both in bulbs and bolts. These findings indicated that the DPPH, FRAP, and CUPRAC methods were stable and reliable in measuring antioxidant capacities of garlic. The correlation between HRSC scavenging activities and polyphenolic compounds was weak, which could be explained by the fact that the presence of other non-flavanol compounds had a strong influence on overall scavenging activity.

**Table 3:** Correlation coefficients of antioxidant capacity (DPPH, HRSC, FRAP, CRAC, MCA), total phenols (TPC), total flavonoids (TFAC) in garlic bulbs.

	TPC	TFAC	DPPH	HRSC	FRAP	CRAC	MCA
TPC	1.0000						
TFAC	0.81**	1.0000					
DPPH	0.51**	0.67**	1.0000				
HRSC	−0.0100	0.1100	−0.0800	1.0000			
FRAP	0.80**	0.85**	0.50**	0.0800	1.0000		
CRAC	0.74**	0.82**	0.71**	0.0800	0.76**	1.0000	
MCA	−0.68**	−0.59**	−0.58**	0.2000	−0.52**	−0.62**	1.0000

**P<0.01;

*P<0.5.

**Table:** Table 4. Correlation coefficients of antioxidant capacity (DPPH, HRSC, FRAP, CRAC, MCA), total phenols (TPC), total flavonoids (TFAC) in garlic bolts.

	TPC	TFAC	DPPH	HRSC	FRAP	CRAC	MCA
TPC	1.0000						
TFAC	0.35*	1.0000					
DPPH	0.68**	0.2500	1.0000				
HRSC	0.2500	−0.35*	0.1000	1.0000			
FRAP	0.92**	0.1600	0.70**	0.35*	1.0000		
CRAC	0.92**	0.3300	0.75**	0.0700	0.85**	1.0000	
MCA	0.2300	−0.0800	0.52**	0.35*	0.34*	0.2200	1.0000

**P<0.01;

*P<0.5.

### In vitro Protection against tertiary butyl hydroperoxide induced cytotoxicity and ROS formation in Human Vascular Endothelial Cells (Oxidative Stress Model)

The bulb extracts of eight garlic cultivars (extract concentrations 1 mg/mL) were tested and compared for cytoprotection against oxidative stress cell death and ROS formation in Human Vascular Endothelial Cells. It showed that all the bulb extracts tested successfully prevented cell death and the order of protection against cell death was, from most protective to least protective after 3 hours incubation: ‘74-X’ extract > ‘Hanzhong Purple’ extract  =  ‘Suzhou white’ extract > ‘Ningqiang mountain garlic’ extract> ‘Cangshan’ extract  =  ‘Russian garlic’ extract > ‘No. 105 from Korea’ extract  =  ‘No. 97 from Guizhou’ extract (Table 5). When tested against ROS formation in Human Vascular Endothelial Cells, all test garlic extracts significantly prevented ROS formation, in the order of most protective to least protective: ‘Cangshan’ (53%)>‘74-x’ (49%)> ‘Hanzhong Purple’ (47%)> ‘Suzhou white’ (46%)> ‘Ningqiang mountain garlic’ (44%)> ‘Russian garlic’ (42%)> ‘No. 97 from Guizhou’ (35%)> ‘No. 105 from Korea’ (25%). In these two experiments with Human Vascular Endothelial Cells, it was evident that the ‘74-x’ and ‘Hanzhong Purple’ extracts had superior protective effects overall when compared to the T-BHP extracts of each garlic cultivars, with the ‘No. 105 from Korea’ extract having the minimal protection. The order of protection of garlic bulb extracts against oxidative stress cell death in Human Vascular Endothelial Cells was almost the same order of preventing ROS formation except for ‘Cangshan’ extract probably because cultivar ‘Cangshan’ had the higher TPC and TFAC in these test garlic cultivars.

**Table 5:** Garlic Extracts Prevent Prevent Cell Death Human Vascular Endothelial Cells (ECV 304).

Compounds added	Percent Cytotoxicity (% trypan blue uptake)			ROS formation (FI units)		
	3 hours			3 hours		
Control	5	±	2	224	±	3.94
+ Tertiary butyl hydroperoxide 1 mM	90	±	2a)	300	±	5.03a)
+ 74-x (1 mg/mL)	55	±	2b)	153	±	6.28b)
+ ‘Ningqiang mountain garlic’ (1 mg/mL)	64	±	3b)	167	±	5.34 b)
+‘Suzhou white’ (1 mg/mL)	59	±	2b)	161	±	6.12 b)
+ ‘Russian garlic’ (1 mg/mL)	66	±	3 b)	174	±	5.00 b)
+ ‘No. 97 from Guizhou’ (1 mg/mL)	71	±	1b)	195	±	5.00b)
+ ‘Hanzhong Purple’ (1 mg/mL)	59	±	2 b)	158	±	6.00 b)
+ ‘No. 105 from Korea’ (1 mg/mL)	71	±	1 b)	226	±	5.00 b)
+‘Cangshan’ (1 mg/mL)	66	±	3b)	141	±	6.00b)
+ VE (50 uM)	61	±	1 b)	177	±	5.86b)
+ ‘Suzhou white’ (1 mg/mL)	7	±	2	214	±	5.16
+ 74-x (1 mg/mL)	5	±	2	191	±	6.00a)
+ ‘Hanzhong Purple’ (1 mg/mL)	4	±	2	195	±	6.00a)

Human umbilical vein endothelial cells (106 cells/mL) were cultured in Dulbecco's modified Eagle's medium (DMEM). ROS was determined by measuring fluorescent intensity of DCF through DCFH-DA penetration. Means ±SE for three separate experiments are given. All extracts are 1 mg/mL. a) Significant as compared to control (p<0.05), b) Significant as compared to tertiary butyl hydroperoxide 1 mM (p<0.05).

Actually, the order of protection of garlic bulb extracts against oxidative stress cell death was almost the same tendency of TPC and TFAC in these test cultivars even the antioxidant capacity. The TPC order was ‘74-X’> ‘Hanzhong Purple’ >‘Ningqiang mountain garlic’ > ‘Cangshan’ > ‘No. 97 from Guizhou’ > ‘Russian garlic’ > ‘No. 105 from Korea’ > ‘Suzhou white’; and the TFAC of these test cultivars was ‘74-X’> ‘Hanzhong Purple’ > ‘Ningqiang mountain garlic’ > ‘No. 97 from Guizhou’ > ‘Cangshan’ > ‘Suzhou white’ > ‘Russian garlic’ > ‘No. 105 from Korea’. It was showed that protection capability of these garlic bulb extracts against oxidative stress cell death was consistent with their phenolic content and antioxidant capacity. Due to the variety of polyphenolic compounds found in garlic, it is likely that multiple protective mechanisms may act against oxidative and carbonyl induced cytotoxicity in *in vitro* models. One possible mechanism whereby garlic extracts may decrease oxidative stress in cells is in the prevention of lipid peroxidation[36].

## Conclusions

On the basis of PCA, the garlic cultivars examined in this study could be divided into 3 groups. Group 1 contained 23 cultivars with stronger HRSC and FRAP, as well as higher flavonoid contents. The second group consisted of 16 cultivars with a higher TPC and MCA, and the third group consisted of 4 cultivars with stronger HRSC and FRAP. In addition, significant correlations among different antioxidant assays were observed in both bolts and bulbs. These antioxidant properties were highly correlated with the presence of the primary phenolic compounds. It was showed that the bulb extracts of eight test garlic cultivars successfully prevented Human Vascular Endothelial cell death and ROS formation, in which cultivar ‘74-x’ and ‘Hanzhong purple’ had superior protective effects and cultivar ‘No. 105 from Korea’ had the lower protection capability against cell death and ROS formation, which was consistent with their phenolic content and antioxidant capacity.
